# Supplementary material for: Do Comorbidities and Daily Medication before SARS-CoV-2 Infection Play a Role in Self-Reported Post-Infection Symptoms?
Source: J Clin Med. 2022 Oct 25;11(21):6278. doi: 10.3390/jcm11216278 (PMC9657459; doi:10.3390/jcm11216278)
Supplement: Supplementary file 1 [file jcm-11-06278-s001.zip › jcm-1912479-supplementary.pdf]

Journal of
Clinical Medicine

Supplementary material S1.

Links to websites where the information about the study was distributed.

https://www.kaunoklinikos.lt/apie-mus/naujienos/covid-19-liga-kokie-pozymiai-ir-liekamieji-reiskiniai-labiausiai-vargina-lietu-vius.html

https://www.facebook.com/groups/Virusas/?multi_permalinks=648288256333847

https://www.facebook.com/groups/1994091020875362/?multi_permalinks=3124472811170505 (closed)

https://www.facebook.com/groups/159021887526863

https://www.facebook.com/groups/412929278885515/?multi_permalinks=2097940567051036 (closed)

https://www.facebook.com/groups/3495414077220547

https://www.facebook.com/groups/lietuvos.mamyciu.turgelis

https://www.facebook.com/groups/1312138168987450

https://www.facebook.com/groups/371615077313631

Supplementary Figure S1. Participants of survey.

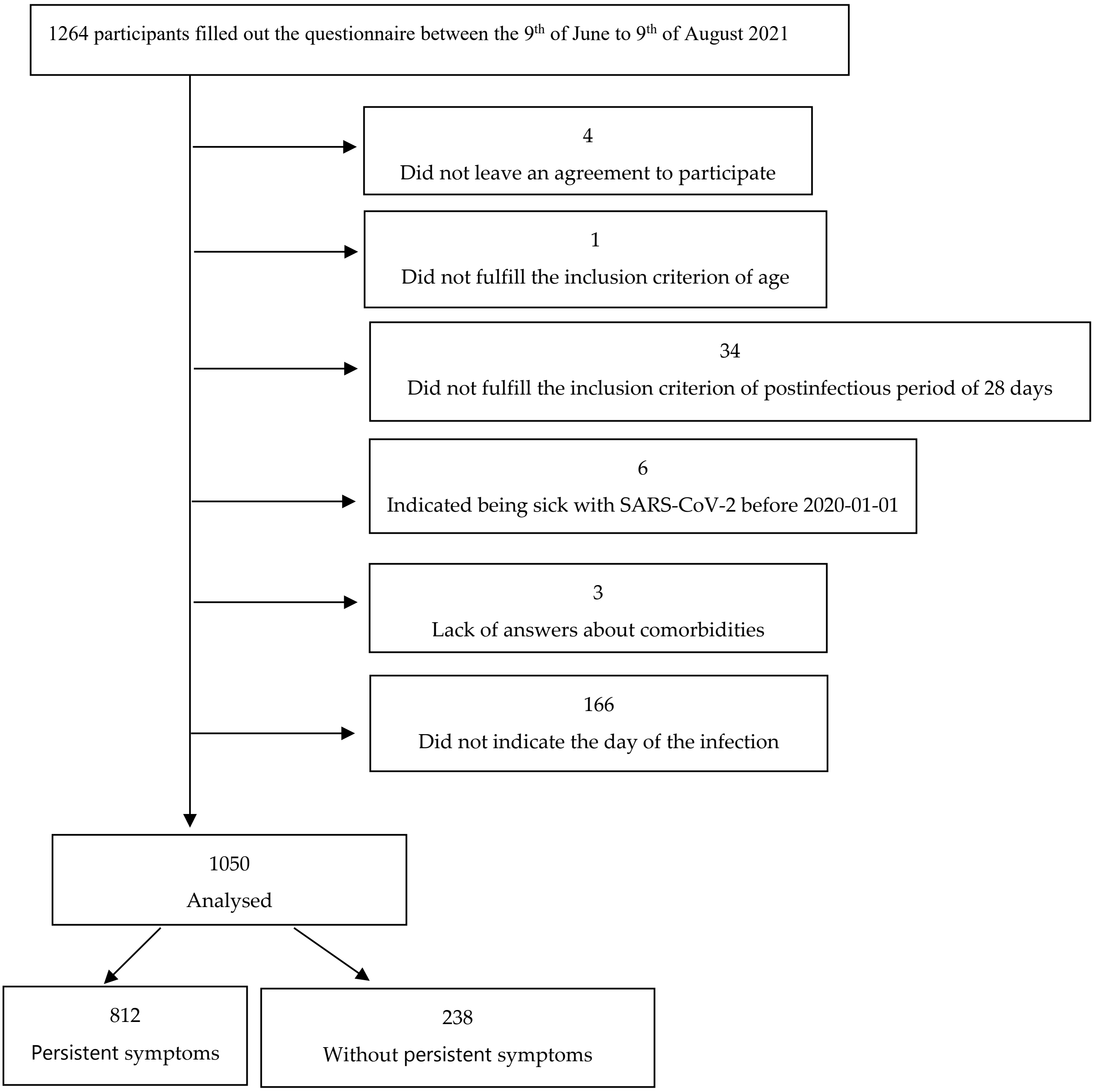

Supplementary Table S1. Disorders presented as the number of participants as well as percentage of the whole population (in brackets) indicating one or more disorders. Disorders are presented as grouped and as indicated in the questionnaire. "Indicating" means the number of indicated disorders (more than 1) in the questionnaire. The total population was 1,050 participants.

Disorders indicated in the questionnaire	Number of participants indicating each disorder
Cardiovascular (6)<br><br>1. High blood pressure / arterial hypertension<br>2. Other cardiovascular diseases<br>3. Chronic ischemic heart disease<br>4. Arrhythmia<br>5. Tachycardia<br>6. POTS	194 (19%)<br><br>153 (15%)<br>59 (6%)<br>2 (0.2%)<br>1 (0.1%)<br>1 (0.1%)<br>1 (0.1%)<br><br>Indicating 2 cardiovascular disorders N= 23 (2%)
Endocrine (3)<br><br>7. Obesity<br>8. Other metabolic diseases<br>9. Diabetes	121 (12%)<br><br>96 (9%)<br>25 (2%)<br>21 (2%)<br><br>Indicating 2 endocrinedisorders N=17 (2%)<br>Indicating 3 endocrine disorders N=2 (0.2%)
Thyroidea-related (5)<br><br>10. Hypothyroidism<br>11. Thyroid diseases<br>12. Autoimmune thyroiditis<br>13. Thyrotoxicosis<br>14. Nodular goitre	82 (8%)<br><br>53 (5%)<br>25 (2%)<br>2 (0.2%)<br>1 (0.1%)<br>1 (0.1%)
Neurological (3)<br><br>15. Neurological diseases<br>16. Sleep disorders<br>17. Epilepsy	103 (10%)<br><br>65 (6%)<br>48 (5%)<br>1 (0.1%)<br><br>Indicating 2 neurological disorders N=12 (1%)
Psychiatric (4)	74 (7%)

18. Anxiety<br>19. Depression<br>20. Other psychiatric disorders<br>21. Bipolar syndrome	55 (5%)<br>31 (3%)<br>5 (0.5%)<br>1 (0.1%)<br><br>Indicating 2 psychiatric disorders N=14 (1%)<br>Indicating 3 psychiatric disorders N=2 (0.2%)
Gastrointestinal (5)<br><br>22. Diseases of the gastrointestinal tract<br>23. Hernia<br>24. Gilbert´s syndrome<br>25. Colitis<br>26. Gallbladder disease	74 (7%)<br><br>69 (7%)<br>1 (0.1%)<br>2 (0.2%)<br>1 (0.1%)<br>1 (0.1%)
27. Allergic diseases	71 (7%)
Pulmonary (3)<br><br>28. Asthma<br>29. Other lung diseases<br>30. Diseases of the upper respiratory tract	53 (5%)<br><br>34 (3%)<br>15 (1%)<br>7 (0.7%)<br><br>Indicating 2 pulmonary disorders N=3 (0.3%)
Inflammatory rheumatic (2)<br><br>31. Rheumatic diseases<br>32. Rheumatoid arthritis	34 (3%)<br><br>33 (3%)<br>1 (0.1%)
Chronic pain (3)<br><br>33. Chronic pain syndrome<br>34. Migraine<br>35. Other chronic pain	32 (3%)<br><br>25 (2%)<br>6 (0.6%)<br>1 (0.1%)
Haematological (2)<br><br>36. Anaemia<br>37. Blood clotting disorders	19 (2%)<br><br>12 (1%)<br>7 (0.7%)
38. Oncological diseases	18 (2%)
Dermatological (3)	18 (2%)

39. Skin diseases<br>40. Psoriasis<br>41. Vertigo syndrome	12 (1%)<br>5 (0.5%)<br>1 (0.1%)
42. Kidney disease	11 (1%)
Gynaecological (3)<br>43. Gynaecological diseases<br>44. Polycystic ovary syndrome<br>45. Endometriosis	10 (0.9%)<br>8 (0.8%)<br>1 (0.1%)<br>1 (0.1%)<br>Indicating 2 gynaecological disorders N=1 (0.1%)
46. Immunodeficiency diseases	6 (0.6%)
Others (3):<br>47. Autoimmune diseases<br>48. Glaucoma<br>49. Otosclerosis	3 (0.3%)<br>1 (0.1%)<br>1 (0.1%)<br>1 (0.1%)

Supplementary Table S2. Persistent symptoms in 1,050 participants presented as numbers and percentages. The data show all persistent symptoms regarding intake of medication prior acute SARS-CoV-2 infection. Chi-square test between the groups, p value lower than 0.05 is indicated in bold style.

	Participants indicating persistent symptoms	Reported no medication prior to acute infection N, (%)	Reported medication prior to acute infection N, (%)	p-value between the groups
1. Reduced physical capacity	423 (40.3%)	291 (38.2 %)	132 (45.8 %)	**p=0.015**
2. Common fatigue	405 (38.6%)	271 (35.6 %)	134 (46.5 %)	**p<0.001**
3. Memory problems	354 (33.7%)	239 (31.4 %)	115 (39.9 %)	**p=0.006**
4. Concentration disorders	313 (29.8%)	223 (29.3 %)	90 (31.3 %)	p=0.289
5. Altered sense of smell	247 (23.5%)	194 (25.5 %)	53 (18.4 %)	**p=0.009**
6. Headache	209 (19.9%)	139 (18.2 %)	70 (24.3 %)	**p=0.019**

7. Heart palpitations / tachycardia	207 (19.7%)	132 (17.3 %)	75 (26 %)	**P=0.001**
8. Unstable mood	199 (19%)	146 (19.2 %)	53 (18.4 %)	p=0.427
9. Hair loss	183 (17.4%)	109 (14.1 %)	74 (25.4 %)	**p<0.001**
10. Insomnia	181 (17.2%)	124 (16.3 %)	57 (19.8 %)	p=0.106
11. Shortness of breath	158 (15%)	100 (13.1 %)	58 (20.1 %)	**p<0.004**
12. Pain floating around	154 (14.7%)	100 (13. %)	54 (18.8 %)	**p=0.015**
13. Anxiety	149 (14.2%)	105 (13.8 %)	44 (15.3 %)	p=0.298
14. Dizziness	140 (13.3%)	89 (11.7 %)	51 (17.7 %)	**p<0.008**
15. Altered sense of taste	126 (12%)	101 (13.3 %)	25 (8.7 %)	**p=0.025**
16. Increased sleepiness	125 (11.9%)	92 (12.1 %)	33 (11.5 %)	p=0.438
17. Sweating night-time	111 (10.6%)	66 (8.7 %)	45 (15.6 %)	**P=0.001**
18. Muscle/bone/joint pain	105 (14.7%)	57 (7.5 %)	48 (16.7 %)	**p<0.001**
19. Pain/pressure in chest	97 (9.2%)	62 (8.1 %)	35 (12.2 %)	**p=0.032**
20. Sensation of head pressure	92 (8.8%)	61 (8.0 %)	31 (10.8 %)	p=0.1
21. Dry eyes	84 (8%)	55 (7.2 %)	29 (10.1 %)	p=0.084
22. Increased weight	84 (8%)	45 (5.9 %)	39 (13.5 %)	**p<0.001**
23. Blurred vision	78 (7.4%)	43 (5.6 %)	35 (12.2 %)	**p<0.001**
24. Increased blood pressure	76 (7.2%)	46 (6 %)	30 (10.4 %)	**p=0.012**
25. Low back pain	75 (7.1%)	40 (5.2 %)	35 (12.2 %)	**p<0.001**
26. Cramps in muscles	72 (6.9%)	41 (6.8 %)	31 (10.8 %)	**P=0.002**
27. Dry skin	72 (6.9%)	47 (7.5 %)	25 (5.8 %)	p=0.098
28. Balance problems	68 (6.5%)	50 (6.6 %)	18 (6.3 %)	p=0.49
29. Other sleep problems	65 (6.2%)	47 (6.2 %)	18 (6.3 %)	p=0.53
30. Dry cough	62 (5.9%)	40 (5.2 %)	22 (7.6 %)	p=0.096
31. Reflux in stomach	60 (5.7%)	30 (3.9 %)	30 (10.4 %)	**p<0.001**
32. Lump in throat	59 (5.6%)	36 (4.7 %)	23 (8.0 %)	**p=0.032**
33. Cold feet/numbness/pain	59 (5.6%)	29 (3.8 %)	30 (10.4 %)	**p<0.001**
34. Fear	57 (5.4%)	34 (4.5 %)	23 (8.0 %)	**p=0.021**
35. Blinking in the eyes	55 (5.2%)	36 (4.7 %)	19 (6.6 %)	p=0.145
36. Cold hands/numbness/pain	54 (5.1%)	32 (4.2 %)	22 (7.6 %)	**p=0.021**
37. Varying symptoms	54 (5.1%)	38 (5.0 %)	16 (5.6 %)	p=0.407
38. Pain in thoracic spine/neck	52 (5%)	26 (3.4 %)	26 (9.0 %)	**p<0.001**
39. Hearing impairment (growl / tinnitus)	48 (4.6%)	28 (3.7 %)	20 (6.9 %)	**P=0.021**
40. Lack of appetite	47 (4.5%)	35 (4.6 %)	12 (4.2 %)	p=0.457
41. Clogged / running nose	47 (4.5%)	36 (4.7 %)	11 (3.8 %)	p=0.328
42. Great / constant thirst	45 (4.3%)	30 (3.9 %)	15 (5.2 %)	p=0.227
43. Weight loss	44 (4.2%)	29 (3.8 %)	15 (5.2 %)	p=0.199
44. Various skin rashes	40 (3.8%)	28 (3.7 %)	12 (4.2 %)	p=0.415
45. Cough / sputum	40 (3.8%)	25 (3.3 %)	15 (5.2 %)	p=0.103
46. Other neuralgic pains	36 (3.4%)	20 (2.6 %)	16 (5.6 %)	**p=0.019**
47. Nausea	33 (3.1%)	21 (2.8 %)	12 (4.2 %)	p=0.165
48. Anaemia	28 (2.7%)	18 (2.4 %)	10 (3.5 %)	p=0.214

49. Painful / sensitive scalp	28 (2.7%)	14 (1.8 %)	14 (4.9 %)	**P=0.008**
50. Mouth ulcers / ulcers	28 (2.7%)	13 (1.7 %)	15 (5.2 %)	**p=0.003**
51. Diarrhoea / constipation	24 (2.3%)	14 (1.8 %)	10 (3.5 %)	p=0.092
52. Sore throat	23 (2.2)	15 (2.0 %)	8 (2.8 %)	p=0.279
53. Prolonged fever	21 (2%)	16 (2.1 %)	5 (1.7 %)	p=0.463
54. Unusually low body temperature	18 (1.7%)	14 (1.8 %)	4 (1.4 %)	p=0.423
55. Low blood pressure	18 (1.7%)	11 (1.4 %)	7 (2.4 %)	p=0.199
56. Inflammations/infections in other organs	16 (1.5%)	6 (0.8 %)	10 (3.5 %)	**P=0.003**
57. Inflammation in the eyes / barley	13 (1.2%)	9 (1.2 %)	4 (1.4 %)	p=0.497
58. Chills without fever	11 (1%)	7 (0.9 %)	4 (1.4 %)	p=0.355
59. Fever / chills	10 (1%)	8 (1 %)	2 (0,7 %)	p=0.453
60. Altered salivation	9 (0.9%)	5 (0.7 %)	4 (1.4 %)	p=0.213
61. Renal pain/difficulty urinating	8 (0.8%)	7 (0.9 %)	1 (0.3 %)	p=0.309
62. Vomiting	2 (0.2%)	2 (0.3 %)	0 (0 %)	p=0.526
